# The functional expression of extracellular calcium-sensing receptor in rat pulmonary artery smooth muscle cells

**DOI:** 10.1186/1423-0127-18-16

**Published:** 2011-02-11

**Authors:** Guang-wei Li, Qiu-shi Wang, Jing-hui Hao, Wen-jing Xing, Jin Guo, Hong-zhu Li, Shu-zhi Bai, Hong-xia Li, Wei-hua Zhang, Bao-feng Yang, Guang-dong Yang, Ling-yun Wu, Rui Wang, Chang-qing Xu

**Affiliations:** 1 Department of Pathophysiology, Qiqihar Medical University, Qiqihar 161006, PR China; 2 Department of Pathophysiology, Harbin Medical University, Harbin 150086, PR China; 3 Department of Pharmacology, Harbin Medical University, Harbin 150086, PR China; 4 Bio-pharmaceutical Key Laboratory of Heilongjiang Province, Harbin 150086, PR China; 5 The second affiliated hospital of Harbin Medical University, Harbin 150086, PR China; 6 Department of Biology, Lakehead University, Thunder Bay, Ont., P7B5E1, Canada

## Abstract

**Background:**

The extracellular calcium-sensing receptor (CaSR) belongs to family C of the G protein coupled receptors. Whether the CaSR is expressed in the pulmonary artery (PA) is unknown.

**Methods:**

The expression and distribution of CaSR were detected by RT-PCR, Western blotting and immunofluorescence. PA tension was detected by the pulmonary arterial ring technique, and the intracellular calcium concentration ([Ca^2+^]_i_) was detected by a laser-scanning confocal microscope.

**Results:**

The expressions of CaSR mRNA and protein were found in both rat pulmonary artery smooth muscle cells (PASMCs) and PAs. Increased levels of [Ca^2+^]_o _(extracellular calcium concentration) or Gd^3+ ^(an agonist of CaSR) induced an increase of [Ca^2+^]_i _and PAs constriction in a concentration-dependent manner_. _In addition, the above-mentioned effects of Ca^2+ ^and Gd^3+ ^were inhibited by U73122 (specific inhibitor of PLC), 2-APB (specific antagonist of IP_3 _receptor), and thapsigargin (blocker of sarcoplasmic reticulum calcium ATPase).

**Conclusions:**

CaSR is expressed in rat PASMCs, and is involved in regulation of PA tension by increasing [Ca^2+^]_i _through G-PLC-IP_3 _pathway.

## Background

Intracellular calcium, a secondary messenger, plays a key role in various physiological processes. Multiple studies have shown that extracellular calcium can act as a first messenger through the calcium-sensing receptor (CaSR) in various cells [1]. The CaSR belongs to the C family of G protein coupled receptors which was first cloned from bovine parathyroid gland by Brown *et al *[2]. The CaSR is important in maintaining and regulating mineral ion homeostasis. Increasing evidence has indicated that CaSR was functionally expressed in the cardiovascular system. Wang *et al *showed that CaSR was expressed in cardiac tissues and cardiomyocytes, and the activity of CaSR could be regulated by extracellular calcium and spermine [3]. CaSR is also expressed in vascular smooth muscle cells (SMCs). Wonneberger *et al *[4] and Ohanian *et al *[5] demonstrated that CaSR was involved in the regulation of myogenic tone in the gerbil spiral modiolar artery and in rat subcutaneous arteries. Recent study reported that stimulation of CaSR led to up-regulation of VSMC proliferation, and CaSR-mediated PLC activation was important for VSMC survival [6].

Whether the CaSR is expressed in pulmonary artery smooth muscle cells (PASMCs) and its function in PASMCs are unknown. There is marked difference between systemic and pulmonary circulation in physiological and pathophysiological conditions. For example, coronary artery is relaxed but pulmonary artery is contracted under hypoxic condition. Pulmonary vasoconstriction and PASMC proliferation may contribute to hypoxic pulmonary hypertension. Thus, the present study investigated the expression of CaSR in PAMSCs as well as the effect of CaSR activation on pulmonary artery tension in order to provide an experimental basis for the mechanism of pulmonary hypertension involved by CaSR.

## Methods

### Cell preparation and culture

Primary cultures of PASMCs were prepared as previously described [7-9]. Briefly, PASMCs were obtained from Wistar rat PAs. The isolated distal arterial rings were incubated in Hanks balanced salt solution containing 1.5 mg/ml of collagenase II (Sigma, USA) for 20 min. After incubation, the connective tissue and a thin layer of the adventitia were carefully stripped off with fine forceps, and the endothelium was removed by gently scratching the intimal surface with a surgical blade. The remaining smooth muscles were then digested with 1.0 mg/ml of collagenase II for 120 min at 37°C. The cells were cultured in DMEM supplemented with 20% FBS, penicillin (100 units/ml), streptomycin (100 units/ml), and cultured in a humidified incubator with 5% CO_2 _for 3-5 d at 37°C. The cells with typical hill-and-valley morphology, were prepared for experiments. Passage 3-8 cells at 80% confluence were used in all reported experiments [10]. This protocol was approved by Harbin Medical University (Harbin 150086, China).

### RT-PCR

Total RNA from PASMCs was extracted according to the Trizol reagent (Invitrogen, USA) protocol and redissolved in 20 μl of DEPC water before being stored at -70°C. RNA was spectrophotometrically quantified by measuring the optical density of samples at a wavelength of 260-280 nm. The nucleotide sequences of the primers used (TakaRa Co, Ltd.) were as follows: (1) CaSR: sense 5'-ttcggcatcagctttgtg-3', antisense 5'-tgaagatgatttcgtcttcc-3'; (2) GAPDH: sense 5'-ctcaactacatggtctacatg -3', antisense 5'-tggcatggactgtggtcatgag-3', yielding predicted products of 234 and 420 bp, respectively. RT-PCR was performed according to the RT-PCR kit (Promega, USA) protocol. Cycling conditions were as follows: 35 cycles of denaturation at 94°C for 20 s, annealing at 55°C for 40 s, and polymerization at 72°C for 40 s. Aliquots (5 μL) of PCR reactions were electrophoresed through ethidium bromide-stained 1.2% agarose gels and visualized with ethidium bromide. Identity was confirmed by sequencing (Shanghai Sangon Biological Engineering Technology & Services Co.Ltd.) [11].

### Western blotting analysis

Total proteins of the PASMCs were prepared as previously described [12]. Briefly, cells were washed three times with ice-cold phosphate-buffered saline (PBS) and then incubated in cool protein lysate containing the protease inhibitor phenylmethyl sulfonyl fluoride (PMSF) for 20 min. The cells were centrifuged at 14000 g for 15 min at 4°C to remove nuclei and undisrupted cells. The protein concentration of the supernatant was determined using the Bradford protein assay with BSA as a standard. Pulmonary artery tissues and rat cardiac tissue were homogenized with a polytron homogenizer in cool protein lysate containing the protease inhibitor PMSF for 1 h. Protein samples of 40 μg from different experimental groups were separated by 10% SDS-PAGE and transferred to nitrocellulose membranes by electroblotting (300 mA for 2 h). The membranes were blocked in TBST (137 mM NaCl, 20 mM Tris (pH 7.6), and 0.1% (v/v) Tween 20) containing 5% (w/v) skimmed milk at 37°C for 1 h. The membranes were then incubated overnight at 4°C with antibodies against CaSR and anti-β actin (1:500). The membrane of the negative controls was incubated with the antigen-antibody complex. Primary antibodies (a rabbit polyclonal antibody ) and antigenic peptides were obtained from Santa Cruz Biotechnology Inc. (Santa Cruz, CA).The membranes were incubated with secondary antibody AP-IgG(Promega, USA) diluted 1:5000 in TBST for 1 h at room temperature. Antibody-antigen complexes were detected using Western Blue (Promega, USA).

### Immunofluorescence study

The isolated PASMCs were placed onto coverslips, which were covered in 24-well culture plates with polylysine. After cultured for 72 h at 37°C, the PASMCs were washed with PBS, fixed with 4% formaldehyde in PBS for 10 min, and blocked in 1% BSA for 30 min. The cells were incubated with antibody against CaSR (1:100) or the antigen-antibody complex (Santa Cruz, CA) overnight at 4°C. Then, the cells were incubated with secondary IgG (Santa Cruz, CA) (1:1000) conjugated with fluorescein isothiocyanate (FITC), for 1 h at 37°C and washed in PBS and 0.1% Tween 20. DAPI (4,6-diamidino-2-phenylindole; final concentration of 6 μg/ml, Sigma-Aldrich, USA) was included to label nuclei. Fluorescence images were collected with a fluorescence microscope (Leica, Germany).

The separated pulmonary arteries were submerged in freezing embedding medium (2.5% polyvinyl alcohol) and placed in liquid nitrogen, sliced by a freezing microtome, fixed with acetone for 5 min, washed with PBS for 10 min, and blocked in 1% BSA for 30 min. The pulmonary arteries were stained by immunofluorescence similarly to the isolated PASMCs as described above.

### Fluo-3/AM measurements of [Ca^2+^]_i_

The isolated PASMCs were placed onto coverslips, which were covered in 6-well culture plates with polylysine. After 72 h at 37°C, the PASMCs were washed with PBS and were then incubated with 5 μM Fluo-3/AM for 30 min at 37°C in the dark. The cells were rinsed three times with Tyrode's solution to remove the remaining dye, and they were further incubated in Tyrode's solution or Ca^2+^-free Tyrode's solution. During the experiment, FI (fluorescence intensity) of fluo-3 in PASMCs was recorded using a laser-scanning confocal microscope (Olympus, Japan) with excitation at 488 nm and emission at 530 nm.

Following a 60s baseline recording in 1.8 mM CaCl_2_, CaCl_2 _concentration in the medium was increased gradually from 2.5 to 12.5 mM, and intracellular fluo-3 fluorescence measurements continued for 300s. In another groups, cells were exposed to Ca^2+ ^(10 mM) and Gd^3+ ^(300 μM) and then recorded for 120 s at 3s intervals. In some experiments, the PASMCs preincubated with specific inhibitor, NiCl_2 _(0.1 mM, inhibitor of Na^+^-Ca^2+ ^exchanger) [12,13], CdCl_2 _(0.02 mM, inhibitor of L-type calcium channel) [12,13], NPS2390 (10 μM, antagonist of CaSR)[14,15], U73122 (10 μM, PLC-specific inhibitor) [16,17], U73343(10 μM, U73122 inactive analogue) [17], thapsigargin (10 μM, blocker of sarcoplasmic reticulum calcium-ATPase) [18,19], caffeine (10 mM, depleted agent of the ryanodine receptor-operated Ca^2+ ^store) [18] for 30 min and 2-APB (75 μM, IP_3 _receptor antagonist) [20] for 20 min before Ca^2+ ^(10 mM) and Gd^3+ ^(300 μM) challenge. Image analysis was performed off-line using Fluoview-FV300 (Olympus, Japan) to select cell regions from which FI was extracted, and further analysis was conducted with Excel (Microsoft) and Origin Version 7.5 software (OriginLab Corporation). [Ca^2+^]_i _changes were expressed as fluorescence intensity representing FI and normalized to initial fluorescence intensity (FI_0_) [20].

### Tension studies of pulmonary artery rings

Adult male Wistar rats (200-250 g) were provided by the Experimental Animal Center of Harbin Medical University, which is fully accredited by the Institutional Animal Care and Use Committee. The experiment was carried out according to the published protocols [21-23]. Rats were anesthetized with pentobarbital sodium (50 mg/kg). The chest was opened, and then both the heart and lung were removed and immediately placed in cold Krebs solution (in mM: NaCl 118, KCl 4.7, CaCl_2 _2.5, MgSO_4 _0.57, KH_2_PO_4 _1.2, NaHCO_3 _20, EDTA-Na_2 _0.02 and Glucose 10, pH 7.4). The pulmonary arteries (PAs) were dissected out, cleaned of connective tissue and cut into rings under a dissecting microscope. Microdissected distal PAs were cut into rings of approximately 0.5 to 1.5 mm in diameter and examined for isometric contractile responses as described [21-23]. The rings were attached to tension-measuring devices by tungsten wire hooks. Pulmonary arterial rings were treated with CaCl_2 _or GdCl_3 _(Sigma-Aldrich, USA) at various concentrations, and the ring tensions were recorded. After CaCl_2 _or GdCl_3 _was washed off, all vessels relaxed to baseline level. Afterwards, the vessels were incubated with 10 mM NiCl_2 _(inhibitor of Na^+^-Ca^2+ ^exchanger), 0.2 mM CdCl_2 _(inhibitor of L-type calcium channel), 50 μM thapsigargin (Sigma-Aldrich, USA. blocker of sarcoplasmic reticulum calcium-ATPase), 10 μM NPS2390 (Sigma-Aldrich, USA. antagonist of CaSR), 10 mM caffeine (Sigma-Aldrich, USA, depleted agent of the ryanodine receptor-operated Ca^2+ ^store), 50 μM U73122 (Sigma-Aldrich, USA. PLC-specific inhibitor), 50 μM U73343 (Sigma-Aldrich, USA. U73122 inactive analogue), and 150 μM 2-APB (Sigma-Aldrich, USA. IP_3 _receptor antagonist) for 30 min. They were then exposed to CaCl_2 _or GdCl_3 _at various concentrations again, and finally the ring tensions were recorded. Tension data were relayed from the pressure transducers to a signal amplifier. Data were acquired and analyzed with CODAS software (DataQ Instruments, Inc.).

### Statistical analysis

Statistical analysis was carried out with SAS version 9.1. A two-sided P < 0.05 was considered significant. Continuous variables were expressed as mean ± standard deviation $\bar{X}\pm SD$. The statistical differences between-group were tested with repeated measurement ANOVA.

## Results

### CaSR mRNA expression in rat PASMCs

**Figure 1:**
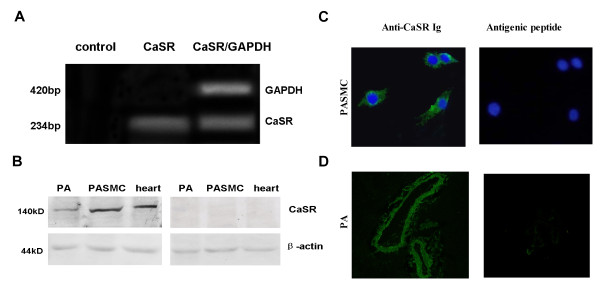
**The calcium sensing receptor (CaSR) is expressed in pulmonary artery smooth muscle cells (PASMCs) and homogenates of pulmonary arteries (PAs)**. A. Detection of CaSR mRNA by RT-PCR in rat PASMCs in the absence or presence of reverse transcriptase and GAPDH. B. Detection of CaSR protein by western blotting in rat cultured PASMCs and PAs. Positive and negative control from rat cardiac tissue (left) and the specific antigenic peptides (right) are also shown. C. Immunofluorescence detection of CaSR in rat PASMCs in the presence of anti-CaSR Ig conjugated with FITC (left) and in the presence of specific antigenic peptides and anti-CaSR Ig (right), (magnification: 400 ×). D. Immunofluorescence detection of CaSR in rat PAs in the presence of anti-CaSR Ig conjugated with FITC (left) and in the presence of specific antigenic peptides and anti-CaSR Ig (right) (magnification: 200 ×), bar = 50 μM.

A cDNA fragment of 234 bp corresponding to the selected CaSR mRNA sequence was detected in PASMCs (Figure 1A). In the absence of reverse transcriptase, no PCR-amplified fragments could be detected, indicating the tested RNA samples were free of genomic DNA contamination. Sequencing results were as follows: ttcggcatcagctttgtgctctgtatctcgtgcatcttggtgaagaccaatcgcgtcctcctggtatttgaagccaagatacccaccagcttccaccggaagtggtgggggctcaacct gcagttcctgctggttttcctctgcaccttcatgcagatcctcatctgcatcatctggctctacacggcgcccccctctagcaccgcaaccatgagctggaagacgaaatcatcttca. The sequence shared 100% identity with the rat CaSR sequence (GenBank/EMBL accession ).

### Protein expression of CaSR in rat PASMCs and PAs

Western blotting with monoclonal CaSR-specific antibody revealed signal of apparent molecular seize of 130 kD in the protein lysates of cultured PASMCs and rat pulmonary artery, consistent with the reported band in cardiac tissue, and there were no bands in the specific antigenic peptides groups (Figure 1B). Immunofluorescence staining showed that CaSR proteins were present in cytoplasm and membrane of the PASMCs (Figure 1C), as well as in rat PAs (Figure 1D). The specific antigenic peptide completely abolished CaSR immunostaining (Figure 1C and 1D).

### Increase in [Ca^2+^]_o _stimulated an increase in [Ca^2+^]_i _via CaSR

**Figure 2:**
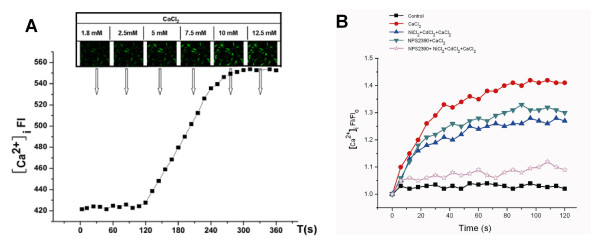
**Effect of different extracellular calcium concentrations ([Ca**^**2+**^**]**_**o**_**), the CaSR antagonist (NPS2390), blocker of L-type calcium channels (CdCl**_**2**_**), and inhibitor of Na**^**+**^**-Ca**^**2+ **^**exchanger (NiCl**_**2**_**) on the intracellular calcium concentrations ([Ca**^**2+**^**]**_**i **_**) in the PAMSCs**. A. [Ca^2+^]_o _from 5 to 12.5 mM caused an increase fluorescent intensities of the [Ca^2+^]_i _in a concentration dependent manner, then we chose 10 mM [Ca^2+^] for the futher experiments (n = 20). B. The cells were exposed to 10 mM Ca^2+^, and FI of [Ca^2+^]_i _was recorded for 120 s. In some experiments, the cells were pre-exposed to 0.1 mM NiCl_2_, 0.02 mM CdCl_2_, and 10 μM NPS2390 for 30 min before Ca^2+ ^challenge.

An initial FI/FI_0 _was regarded as 1.0. As shown in Fig. 2A (n = 20), when [Ca^2+^]_o _increased from 5 to 12.5 mM, FI of [Ca^2+^]_i _was increased in a concentration-dependent manner. Moreover, we also found that 10 mM Ca^2+ ^increased the FI of [Ca^2+^]_i _to 1.297 ± 0.150 at 30 s, 1.357 ± 0.176 at 60 s, 1.402 ± 0.183 at 90 s, and 1.419 ± 0.176 at 120 s in the absence of NiCl_2_, CdCl_2 _and NPS2390_. _The FI of [Ca^2+^]_i _in both the NiCl_2 _+ CdCl_2 _+ CaCl_2 _group and the NPS2390 + CaCl_2 _group was decreased but higher than that in controls (*p *< 0.01 versus control), and the FI of [Ca^2+^]_i _was decreased significantly in the NiCl_2 _+ CdCl_2 _+ NPS2390 + CaCl_2 _group (*p *< 0.01 versus CaCl_2 _group) (Figure 2B, n = 20).

### CaSR activation-induced increase in [Ca^2+^]_i _is dependent on intracellular Ca^2+ ^store in PASMCs

**Figure 3:**
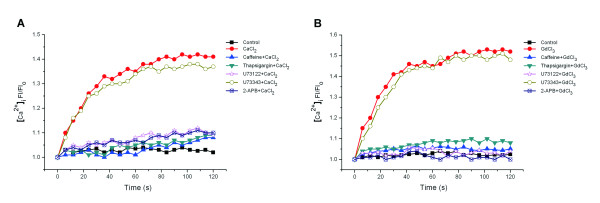
**Effect of various inhibitors on the increase in [Ca**^**2+**^**]**_**i **_**induced by 10 mM [Ca**^**2+**^**]**_**o **_**or 300 μM GdCl**_**3 **_**(CaSR agonists) in PASMCs**. A.10 mM [Ca^2+^]_o _caused an increased FI of [Ca^2+^]_i _( *P *< 0.01 versus control ), and the pretreatment with 10 μM thapsigargin, 10 mM caffeine, 10 μM U73122, or 75 μM 2-APB either decreased or abolish increase the FI of [Ca^2+^]_i _induced by 10 mM [Ca^2+^]_o_, but 10 μM U73343 had no significant effect on it (n = 20). B. The changes in patterns of [Ca^2+^]_i _induced by 300 μM GdCl_3 _and various inhibitors were the same as in A.

Under normal conditions, the increase of intracellular Ca^2+ ^is from extracellular Ca^2+ ^entry and release of intracellular Ca^2+ ^store. To verify that the change in [Ca^2+^]_i _induced by activation of CaSR is dependent on the intracellular Ca^2+ ^store, the PASMCs were incubated with 10 mM caffeine and 10 μM thapsigargin for 30 min, then 10 mM CaCl_2 _or 300 μM GdCl_3 _were added into the media. It was found that Ca^2+ ^FI/FI_0 _was significantly reduced in the presence of caffeine and thapsigargin (*p *< 0.01 versus CaCl_2 _or GdCl_3 _group) (Figure 3A and 3B, n = 20).

### CaSR activation induced an increase in [Ca^2+^]_i _in PASMCs via the PLC-IP_3 _signal transduction pathway

Compared with the 10 mM Ca^2+ ^group, FI/FI_0 _of [Ca^2+^]_i _was decreased in the 2-APB and U73122 pretreated groups. However, U73343 had little effect on [Ca^2+^]_i _FI/FI_0 _(Figure 3A). The treatment with 300 μM Gd^3+ ^also caused a similar response (Figure 3B, n = 20).

### Calcium-induced constriction of pulmonary artery rings

**Figure 4:**
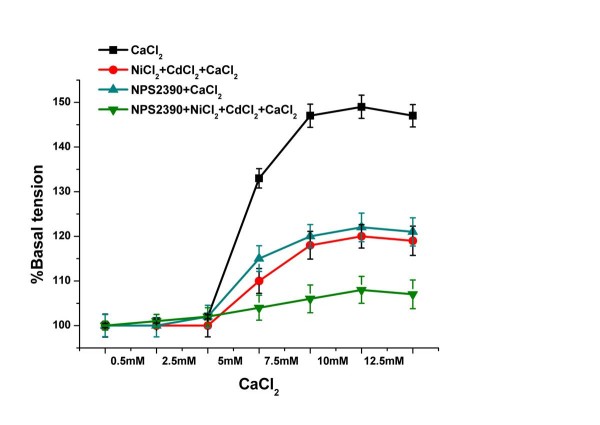
**The effects of different treatments on the vascular tension of the pulmonary arteries with increased [Ca**^**2+**^**]**_**0**_. [Ca^2+^]_o _from 5 to 12.5 mM caused a vasoconstriction of the pulmonary arterys (*P *< 0.01 versus vehicle, n = 8). In the NiCl_2 _+ CdCl_2 _pretreated groups, the vasoconstriction of the pulmonary arterys was attenuated, but it was higher than in the vehicle (*P *< 0.01 versus CaCl_2 _groups). In the NPS2390 pretreated groups, the vasoconstriction of the pulmonary arterys was also attenuated, but it was higher than in the vehicle (*P *< 0.01 versus CaCl_2 _groups). In the NPS2390 + NiCl_2 _+ CdCl_2 _treated groups, the vasoconstriction of the pulmonary artery was significantly attenuated.

An isometric tension of 0.3 g (passive force) was regarded as 100% (vehicle). We observed that an increase in the [Ca^2+^]o from 0.5 to 2.5 mM exerted no effect on tension of the pulmonary artery rings, while increases in [Ca^2+^]o from 5 to 12.5 mM increased vasoconstriction in a dose-dependent manner. In addition, the vasoconstriction was not completely eliminated by NiCl_2_, CdCl_2_, or NPS2390 (Figure 4, n = 8), indicating that [Ca^2+^]_o_-induced vasoconstriction was at least partly mediated via activation of CaSR.

### CaSR activation-induced constriction of pulmonary artery rings is dependent on intracellular Ca^2+ ^store

**Figure 5:**
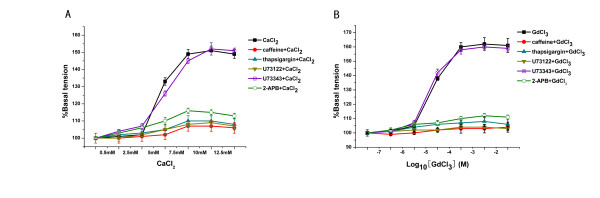
**Effect of various inhibitors on the [Ca**^**2+**^**]**_**o **_**or the [Gd**^**3+**^**]**_**o**_**-induced vasoconstriction**. A. An increase in [Ca^2+^]_o _from 5 to 12.5 mM caused a vasoconstriction of the pulmonary arteries (*P *< 0.01 versus vehicle). In the 10 mM caffeine, 50 μM thapsigargin, 50 μM U73122 or 150 μM 2-APB pretreated groups, the vasoconstriction of the pulmonary arteryies was attenuated, but 50 μM U73343 had no effect on the vasoconstrictions (n = 8). B. [Gd^3+^]_o _from 10^-6 ^to 10^-2 ^M caused similar changes.

We observed that preincubation with 10 mM caffeine or 50 μM thapsigargin for 30 min before Ca^2+ ^and Gd^3+ ^challenge attenuated the constriction of pulmonary artery rings significantly (*p *< 0.01 versus the CaCl_2 _or GdCl_3 _group) (Figure 5A, B. n = 8).

### CaSR activation-induced constriction of pulmonary artery rings via the PLC-IP_3 _signal transduction pathway

Both Ca^2+ ^and Gd^3+ ^evoked increases in tension of pulmonary artery rings in a concentration-dependent manner. U73122 and 2-APB significantly inhibited the constriction of pulmonary artery rings. However, U73343 did not affect the vasoconstriction induced by Ca^2+ ^and Gd^3+ ^(Figure 5A, B. n = 8). Based on these findings, it was speculated that the PLC-IP_3 _signal transduction pathway may be involved in CaSR-induced constriction.

## Discussion

CaSRs are widely expressed in the vessel system, such as in the mesenteric, basilar, renal, coronary [24,25], spiral modiolar arteries[4], subcutaneous vessels [5]and in the aorta[26]. CaSRs are involved in regulation of vascular tension and cell proliferation in these vessels. Increasing evidence indicates that CaSRs play a potential role in vascular calcification and pathogenesis of atherosclerosis, arteriosclerosis and hypertension [27].

Whether the CaSR is expressed in the pulmonary artery has remained unclear. To confirm the existence of CaSRs and its functional expression in some tissues or cells, the following evidence would be necessary. Firstly, CaSR mRNA and protein would be present in the tissue or cells [4]. Secondly, an elevation of [Ca^2+^]_o _would cause an increase of [Ca^2+^]_i_. Thirdly, the [Ca^2+^]_o_-induced increase in [Ca^2+^]_*i *_would be dependent on the release of Ca^2+ ^from thapsigargi- and caffeine-sensitive intracellular stores and dependent on PLC- activation. Fourthly, the CaSR agonists-Gd^3+ ^would cause the same response as an elevation of [Ca^2+^]_o _would [4,28,29].

In this study, comprehensive experiments were carried out, including RT-PCR with CaSR-specific primers, western blotting, and immunofluorescence staining. A cDNA fragment of 234 bp was found in cultured PASMCs, indicating the presence of CaSR mRNA in rat PASMCs. Western blotting analysis showed that CaSR was clearly expressed in rat PASMCs as well as in whole PAs extracts. Heart tissues were used as positive control, and we detected the same size of band (130 kDa) in the lysates of PAMSCs, PAs and heart. There were no bands in specific antigenic peptide groups. However, Ohanian *et al*. reported that immunoblotting of rat subcutaneous artery homogenates with monoclonal CaSR antibody revealed a single immunoreactive band at 159 kDa. This antibody also detected another two bands at 145 and 168 kDa in rat kidney homogenate. CaSR protein is present in human aortic smooth muscle cells, and lysate produces a band 160 kDa [30]. It is generally agreed that bands of 130-170 kDa represent a mature, fully glycosylated form of the CaSR [3,23]. Usually, the band size of CaSR detected by western blotting varies considerably depending on the tissue and cell type, cellular fraction analyzed (membrane or cytosolic), and degree of posttranslational modification (glycosylation) of the CaSR protein [31]. Therefore, the CaSR proteins we detected in rat cultured PASMCs and whole pulmonary artery extract may belong to the mature form of CaSR. Immunofluorescence staining showed that CaSR proteins were observed in vessel walls of PAs and were located in the cytoplasm and plasmalemma of the PASMCs, as shown in other cell types [32,33]. Based on these data, we confirmed the expression of CaSR in PASMCs at the mRNA and protein levels.

To confirm that [Ca^2+^]_o _causes an elevation of [Ca^2+^]_i _mediated by CaSR, Fluo-3/AM was used to measure [Ca^2+^]_i_. The EC50 for Ca^2+ ^activation of CaSR is 3-4 mM [34]. In the present study, it was found that a [Ca^2+^]_o _from 1.8 to 2.5 mM had no effect on [Ca^2+^]_i_, and a [Ca^2+^]_o _from 5 to 12.5 mM induced an elevation of [Ca^2+^]_i _in a concentration-dependent manner_. _This means that in PASMCs, the increase of [Ca^2+^]_o _can cause an elevation of [Ca^2+^]_i_. Additionally, in the presence of NiCl_2 _and CdCl_2, _the FI of [Ca^2+^]_i _has decreased, it is still higher than control group. Furthermore, NPS2390 also decreased the FI of [Ca^2+^]_i_. However, the elevation of [Ca^2+^]_i _induced by 10 mM CaCl_2 _was nearly abolished in the NiCl_2 _+CdCl_2_+NPS2390 group. These results indicated that CaSRs were involved in the elevation of [Ca^2+^]_i _induced by an increased [Ca^2+^]_o, _or that CaSRs at least played a partial role in this process.

In the present study, we found that the pretreatment with caffeine and thapsigargin for 30 min prevented a significant increase of [Ca^2+^]_i _induced by elevated [Ca^2+^]_o _or [Gd^3+^]_o _in PASMCs. It is well known that caffeine is a depletion agent of the ryanodine receptor operated at the Ca^2+ ^store and that thapsigargin is a blocker of sarcoplasmic reticulum calcium ATPase. This suggests that increased [Ca^2+^]_i _induced by CaSR activation is from thapsigargin and caffeine sensitive intracellular Ca^2+ ^stores_._

Wang *et al *reported that elevated [Ca^2+^]_o_, Gd^3+ ^or spermine can cause Ca^2+ ^release from the sarcoplasmic reticulum of rat myocardium via the G protein-PLC-IP_3 _signal transduction pathway [3]. In our experiments, U73122, U73343 and 2-APB were used to reveal the pathway by which CaSR activation causes an increase in [Ca^2+^]_i _in PASMCs. The results showed that, compared with the 10 mM Ca^2+ ^group, the FI/FI_0 _of [Ca^2+^]_i _was markedly decreased in the 2-APB and U73122 pretreated groups. However, preincubation with U73343 did not alter 10 mM [Ca^2+^]_o _-induced elevation of [Ca^2+^]_i_. Pretreatment with 300 μM Gd^3+ ^induced responses similar to those observed in Ca^2+^-treated cultures. These results suggested that activation of CaSR induced the increase in [Ca^2+^]_i _in PASMCs through the PLC-IP_3 _signal transduction pathway.

As we have known, the intracellular Ca^2+^, as an excitation contraction coupling factor, is involved in regulating myocardial contraction and angiotasis. To demonstrate the functional expression of CaSR in PAs, evidence showing that CaSR activation is related to PA tension change needs to be provided. Therefore, we observed the effects of the CaSR agonist, antagonist and other calcium signal-related factors on PAs tension. The results showed that vasoconstriction appeared in a concentration-dependent manner in PAs when [Ca^2+^]_o _was increased from 5 mM to 12.5 mM, and Gd^3+ ^also induced a similar response. In addition, the vasoconstriction was not reversed by an inhibitor of the Na^+^-Ca^2+ ^exchanger and L-type Ca^2+ ^channels, antagonist of CaSR. These findings suggest that an increased [Ca^2+^]_o _or [Gd^3+^]_o _evoked vasoconstriction at least in part by the CaSR. In subcutaneous artery a biphasic response was observed. That is increasing [Ca^2+^]o from 0.5 to 2 mM induced a small vasoconstriction followed by progressive vasodilation from 3 to 10 mM [5]. However, elevation of [Ca^2+^]_o _caused a biphasic vasoconstriction in the spiral modiolar artery [4].

The signal transduction mechanism linked to the CaSR is known to involve the release of Ca^2+ ^from cytosolic stores [35]. Therefore, the PAs were preincubated in caffeine or thapsigargin. We found that caffeine and thapsigargin induced a significant attenuation of the vasoconstriction induced by [Ca^2+^]_o _or [Gd^3+^]_o_, suggesting that [Ca^2+^]_o _or [Gd^3+^]_o _induced constriction of PAs related to the Ca^2+ ^release from thapsigargin and caffeine sensitive intracellular stores.

In the experiment with pulmonary artery rings, we also found that the increases in [Ca^2+^]_o _or [Gd^3+^]_o_-induced PA vasoconstriction were significantly inhibited by U73122 and 2-APB, but not U73343. Thus, the increases in PAs tension induced by Ca^2+ ^and Gd^3+ ^are linked to the PLC-IP_3 _signaling pathway.

## Conclusions

We have demonstrated that functional expression of CaSRs exists in rat PAs and PAMSCs, and that CaSR activation is involved in [Ca^2+^]_i _increase and vasoconstriction through the G-PLC-IP_3 _signal transduction pathway. Pulmonary artery constriction contributes to pulmonary hypertension, so it is expected that CaSR activation could be involved in the development of pulmonary hypertension..

## Competing interests

The authors declare that they have no competing interests.

## Authors' contributions

All authors participated in the design, interpretation of the studies and analysis of the data and review of the manuscript. B-FY, L-YW, RW and C-QX conducted the experiments. C-QX supplied critical reagents. G-WL, Q-SW wrote the manuscript. G-DY and W-hZ finished necessary language corrections to this manuscript. G-WL, Q-sW, J-HH and W-JX are equally contributed.
